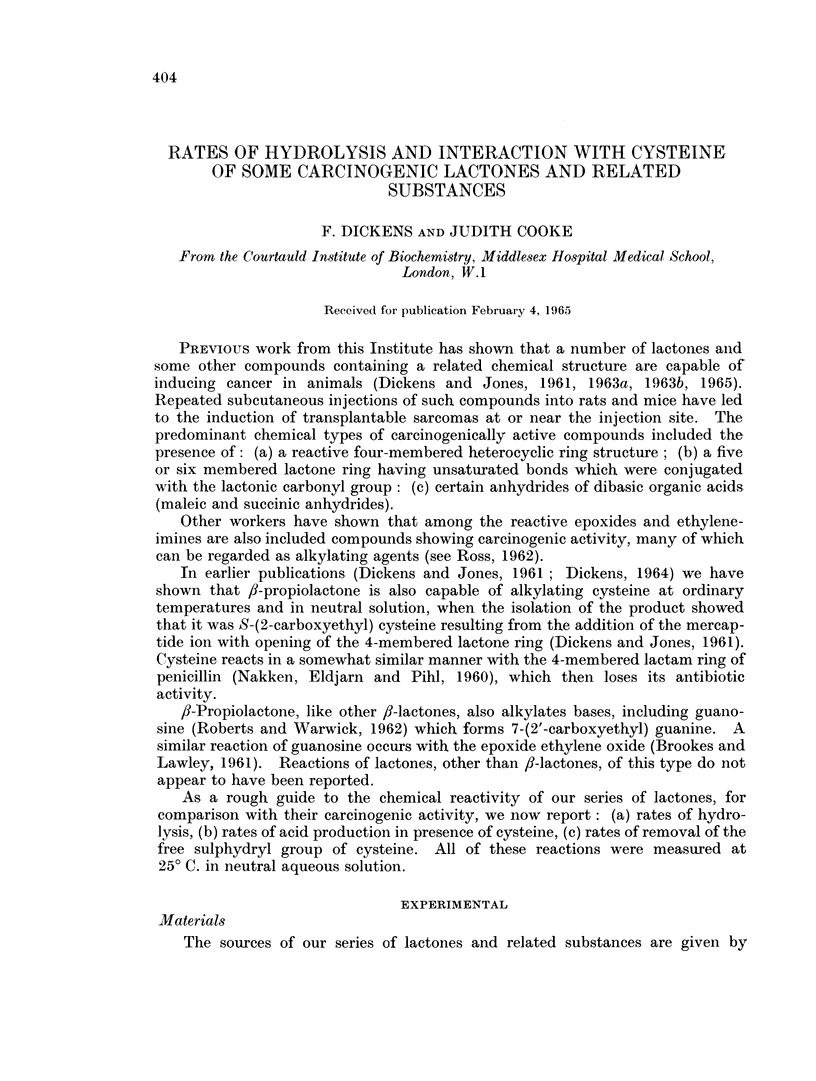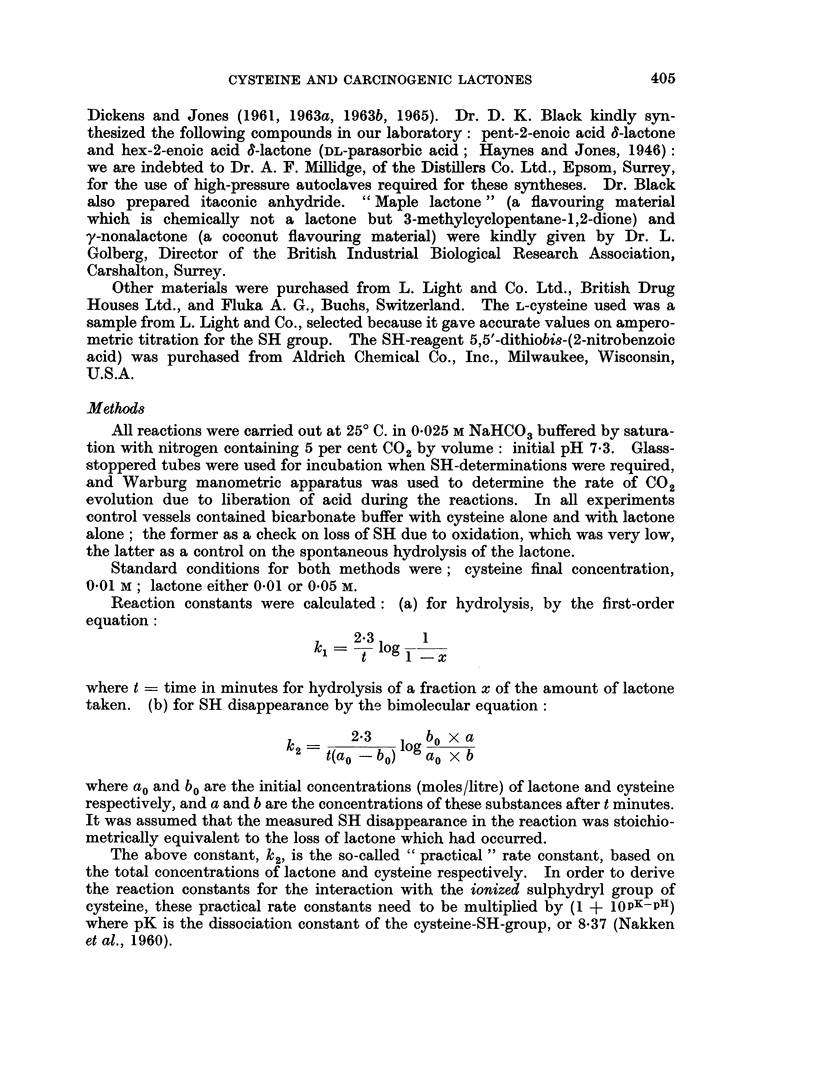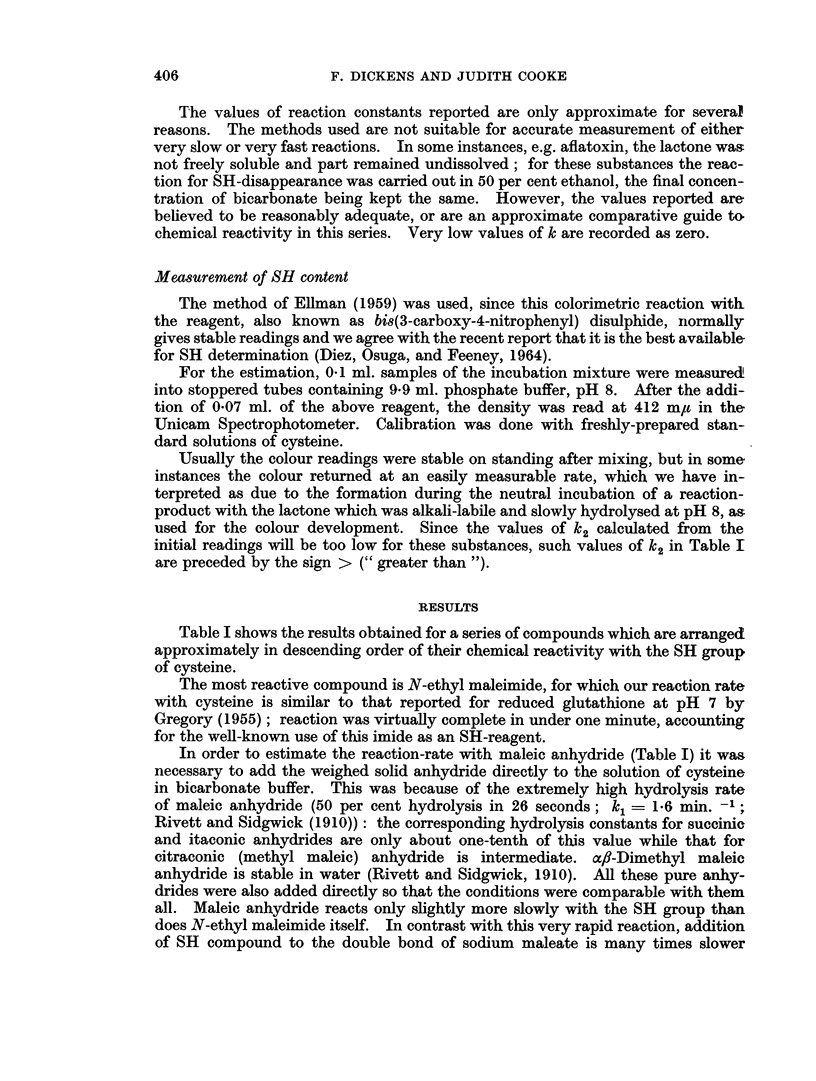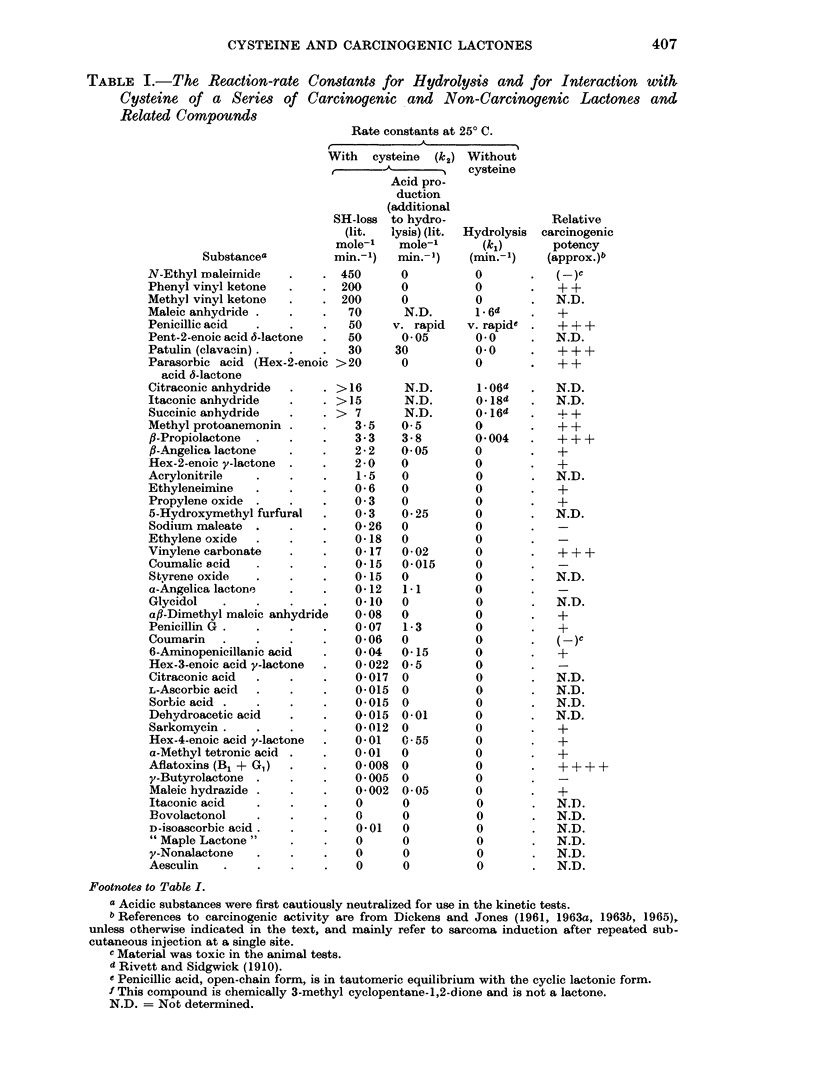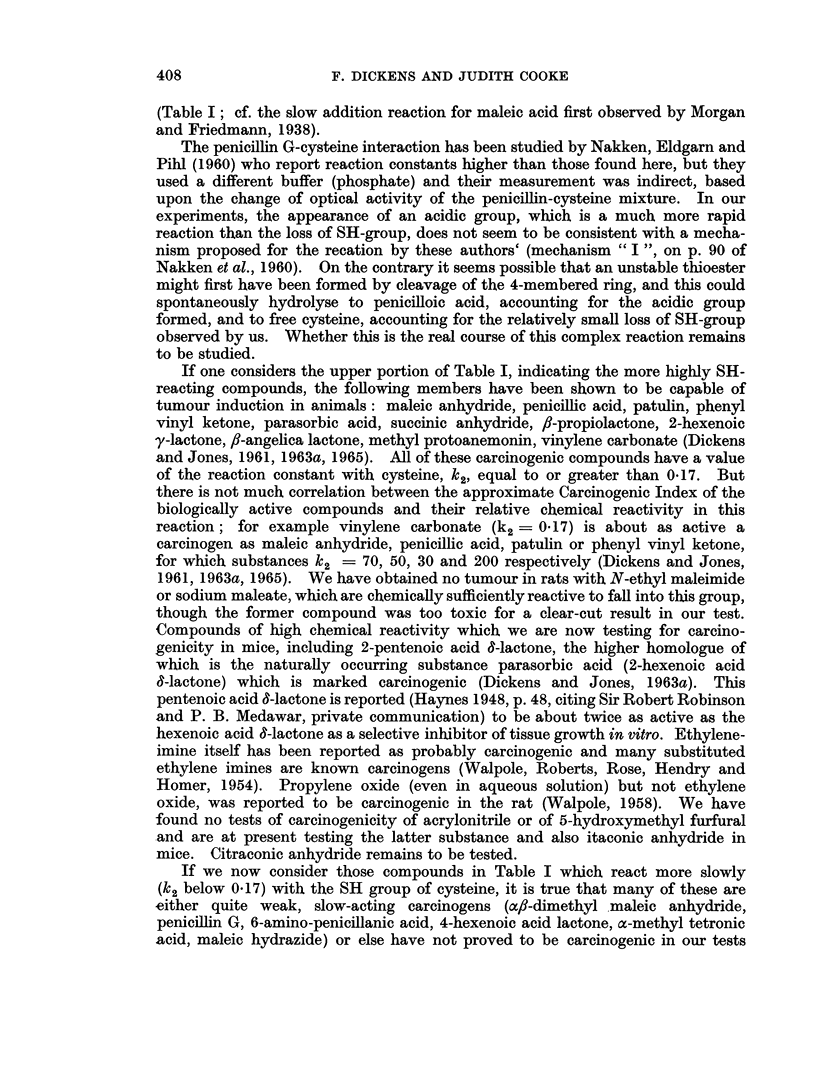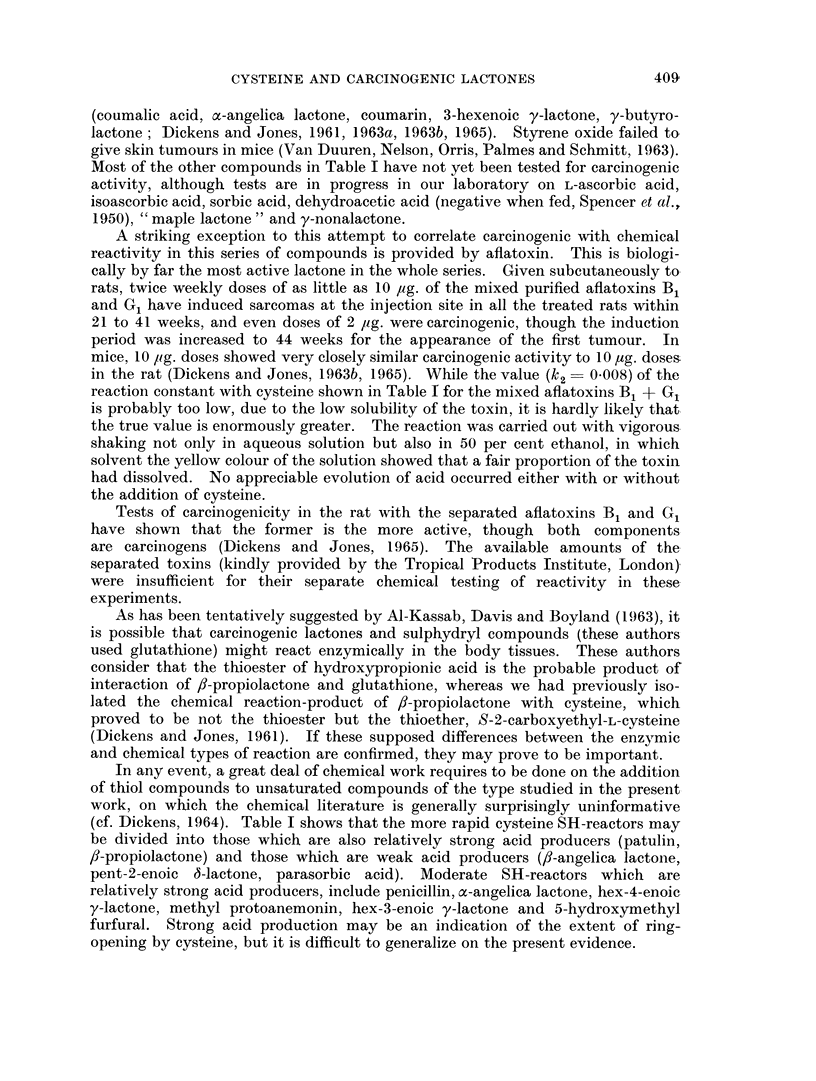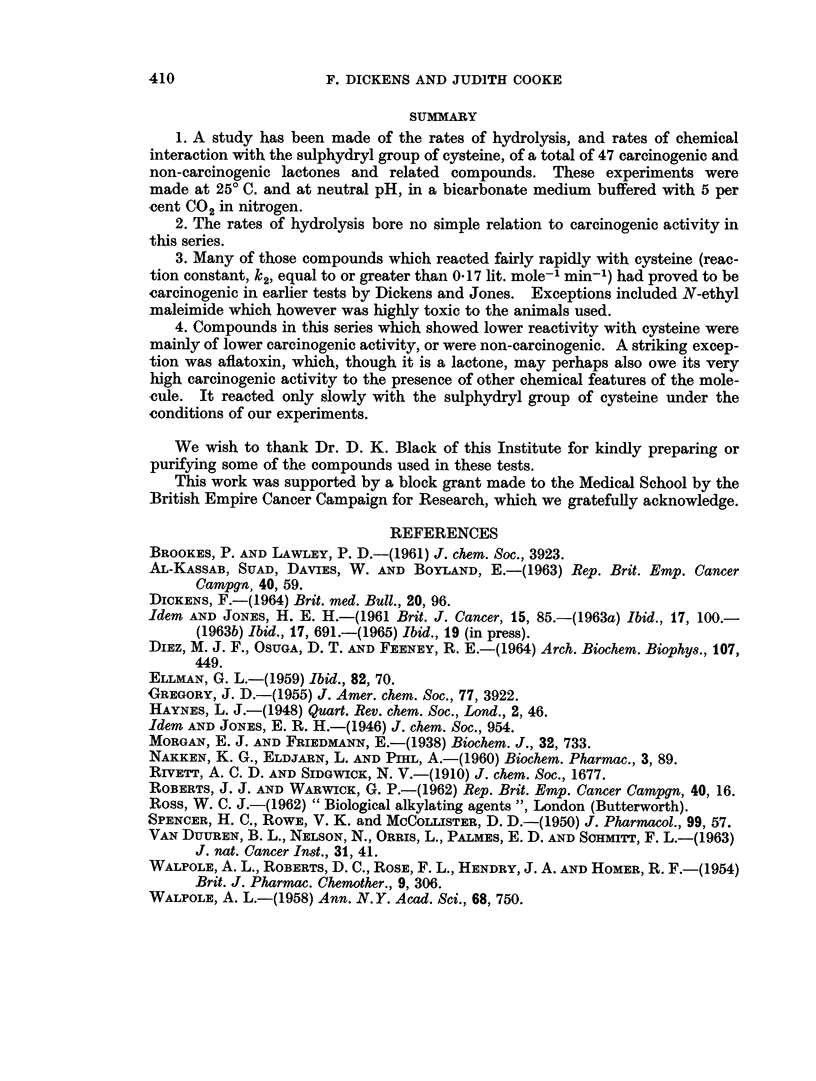# Rates of Hydrolysis and Interaction with Cysteine of some Carcinogenic Lactones and Related Substances

**DOI:** 10.1038/bjc.1965.48

**Published:** 1965-06

**Authors:** F. Dickens, Judith Cooke


### RATES OF HYDROLYSIS AND INTERACTION WITH CYSTEINE OF SOME CARCINOGENIC LACTONES AND RELATED SUBSTANCES

F. DICKENS AND JUDITH COOKE

*From the Courtauld Institute of Biochemistry, Middlesex Hospital Medical School, London, W.1*

Received for publication February 4, 1965

PREVIOUS work from this Institute has shown that a number of lactones and some other compounds containing a related chemical structure are capable of inducing cancer in animals (Dickens and Jones, 1961, 1963*a*, 1963*b*, 1965). Repeated subcutaneous injections of such compounds into rats and mice have led to the induction of transplantable sarcomas at or near the injection site. The predominant chemical types of carcinogenically active compounds included the presence of : (a) a reactive four-membered heterocyclic ring structure ; (b) a five or six membered lactone ring having unsaturated bonds which were conjugated with the lactonic carbonyl group : (c) certain anhydrides of dibasic organic acids (maleic and succinic anhydrides).

Other workers have shown that among the reactive epoxides and ethylene-imines are also included compounds showing carcinogenic activity, many of which can be regarded as alkylating agents (see Ross, 1962).

In earlier publications (Dickens and Jones, 1961 ; Dickens, 1964) we have shown that $\beta$-propiolactone is also capable of alkylating cysteine at ordinary temperatures and in neutral solution, when the isolation of the product showed that it was *S*-(2-carboxyethyl) cysteine resulting from the addition of the mercaptide ion with opening of the 4-membered lactone ring (Dickens and Jones, 1961). Cysteine reacts in a somewhat similar manner with the 4-membered lactam ring of penicillin (Nakken, Eldjarn and Pihl, 1960), which then loses its antibiotic activity.

$\beta$-Propiolactone, like other $\beta$-lactones, also alkylates bases, including guanosine (Roberts and Warwick, 1962) which forms 7-(2′-carboxyethyl) guanine. A similar reaction of guanosine occurs with the epoxide ethylene oxide (Brookes and Lawley, 1961). Reactions of lactones, other than $\beta$-lactones, of this type do not appear to have been reported.

As a rough guide to the chemical reactivity of our series of lactones, for comparison with their carcinogenic activity, we now report : (a) rates of hydrolysis, (b) rates of acid production in presence of cysteine, (c) rates of removal of the free sulphydryl group of cysteine. All of these reactions were measured at 25° C. in neutral aqueous solution.

### EXPERIMENTAL

### *Materials*

The sources of our series of lactones and related substances are given by

Dickens and Jones (1961, 1963*a*, 1963*b*, 1965). Dr. D. K. Black kindly synthesized the following compounds in our laboratory : pent-2-enoic acid $\delta$-lactone and hex-2-enoic acid $\delta$-lactone (DL-parasorbic acid ; Haynes and Jones, 1946) : we are indebted to Dr. A. F. Millidge, of the Distillers Co. Ltd., Epsom, Surrey, for the use of high-pressure autoclaves required for these syntheses. Dr. Black also prepared itaconic anhydride. " Maple lactone " (a flavouring material which is chemically not a lactone but 3-methylcyclopentane-1,2-dione) and $\gamma$-nonalactone (a coconut flavouring material) were kindly given by Dr. L. Golberg, Director of the British Industrial Biological Research Association, Carshalton, Surrey.

Other materials were purchased from L. Light and Co. Ltd., British Drug Houses Ltd., and Fluka A. G., Buchs, Switzerland. The L-cysteine used was a sample from L. Light and Co., selected because it gave accurate values on amperometric titration for the SH group. The SH-reagent 5,5′-dithio*bis*-(2-nitrobenzoic acid) was purchased from Aldrich Chemical Co., Inc., Milwaukee, Wisconsin, U.S.A.

### *Methods*

All reactions were carried out at 25° C. in 0·025 M $NaHCO_3$ buffered by saturation with nitrogen containing 5 per cent $CO_2$ by volume : initial pH 7·3. Glass-stoppered tubes were used for incubation when SH-determinations were required, and Warburg manometric apparatus was used to determine the rate of $CO_2$ evolution due to liberation of acid during the reactions. In all experiments control vessels contained bicarbonate buffer with cysteine alone and with lactone alone ; the former as a check on loss of SH due to oxidation, which was very low, the latter as a control on the spontaneous hydrolysis of the lactone.

Standard conditions for both methods were ; cysteine final concentration, 0·01 M ; lactone either 0·01 or 0·05 M.

Reaction constants were calculated : (a) for hydrolysis, by the first-order equation :

$$k_1 = \frac{2 \cdot 3}{t} \log \frac{1}{1 - x}$$

where $t$ = time in minutes for hydrolysis of a fraction $x$ of the amount of lactone taken. (b) for SH disappearance by the bimolecular equation :

$$k_2 = \frac{2 \cdot 3}{t(a_0 - b_0)} \log \frac{b_0 \times a}{a_0 \times b}$$

where $a_0$ and $b_0$ are the initial concentrations (moles/litre) of lactone and cysteine respectively, and $a$ and $b$ are the concentrations of these substances after $t$ minutes. It was assumed that the measured SH disappearance in the reaction was stoichiometrically equivalent to the loss of lactone which had occurred.

The above constant, $k_2$, is the so-called " practical " rate constant, based on the total concentrations of lactone and cysteine respectively. In order to derive the reaction constants for the interaction with the *ionized* sulphydryl group of cysteine, these practical rate constants need to be multiplied by $(1 + 10^{pK-pH})$ where pK is the dissociation constant of the cysteine-SH-group, or 8·37 (Nakken *et al.*, 1960).

The values of reaction constants reported are only approximate for several reasons. The methods used are not suitable for accurate measurement of either very slow or very fast reactions. In some instances, e.g. aflatoxin, the lactone was not freely soluble and part remained undissolved ; for these substances the reaction for SH-disappearance was carried out in 50 per cent ethanol, the final concentration of bicarbonate being kept the same. However, the values reported are believed to be reasonably adequate, or are an approximate comparative guide to chemical reactivity in this series. Very low values of $k$ are recorded as zero.

*Measurement of SH content*

The method of Ellman (1959) was used, since this colorimetric reaction with the reagent, also known as *bis*(3-carboxy-4-nitrophenyl) disulphide, normally gives stable readings and we agree with the recent report that it is the best available for SH determination (Diez, Osuga, and Feeney, 1964).

For the estimation, 0·1 ml. samples of the incubation mixture were measured into stoppered tubes containing 9·9 ml. phosphate buffer, pH 8. After the addition of 0·07 ml. of the above reagent, the density was read at 412 m$\mu$ in the Unicam Spectrophotometer. Calibration was done with freshly-prepared standard solutions of cysteine.

Usually the colour readings were stable on standing after mixing, but in some instances the colour returned at an easily measurable rate, which we have interpreted as due to the formation during the neutral incubation of a reaction-product with the lactone which was alkali-labile and slowly hydrolysed at pH 8, as used for the colour development. Since the values of $k_2$ calculated from the initial readings will be too low for these substances, such values of $k_2$ in Table I are preceded by the sign $>$ (" greater than ").

### RESULTS

Table I shows the results obtained for a series of compounds which are arranged approximately in descending order of their chemical reactivity with the SH group of cysteine.

The most reactive compound is $N$-ethyl maleimide, for which our reaction rate with cysteine is similar to that reported for reduced glutathione at pH 7 by Gregory (1955) ; reaction was virtually complete in under one minute, accounting for the well-known use of this imide as an SH-reagent.

In order to estimate the reaction-rate with maleic anhydride (Table I) it was necessary to add the weighed solid anhydride directly to the solution of cysteine in bicarbonate buffer. This was because of the extremely high hydrolysis rate of maleic anhydride (50 per cent hydrolysis in 26 seconds ; $k_1 = 1{\cdot}6$ min. $^{-1}$ ; Rivett and Sidgwick (1910)) : the corresponding hydrolysis constants for succinic and itaconic anhydrides are only about one-tenth of this value while that for citraconic (methyl maleic) anhydride is intermediate. $\alpha\beta$-Dimethyl maleic anhydride is stable in water (Rivett and Sidgwick, 1910). All these pure anhydrides were also added directly so that the conditions were comparable with them all. Maleic anhydride reacts only slightly more slowly with the SH group than does $N$-ethyl maleimide itself. In contrast with this very rapid reaction, addition of SH compound to the double bond of sodium maleate is many times slower

TABLE I.—*The Reaction-rate Constants for Hydrolysis and for Interaction with Cysteine of a Series of Carcinogenic and Non-Carcinogenic Lactones and Related Compounds*

| | Rate constants at 25° C. | | | |
|---|---|---|---|---|
| | With cysteine ($k_2$) | | Without cysteine | |
| Substance[a] | SH-loss (lit. mole$^{-1}$ min.$^{-1}$) | Acid production (additional to hydrolysis) (lit. mole$^{-1}$ min.$^{-1}$) | Hydrolysis ($k_1$) (min.$^{-1}$) | Relative carcinogenic potency (approx.)[b] |
| *N*-Ethyl maleimide | 450 | 0 | 0 | (−)[c] |
| Phenyl vinyl ketone | 200 | 0 | 0 | ++ |
| Methyl vinyl ketone | 200 | 0 | 0 | N.D. |
| Maleic anhydride | 70 | N.D. | 1·6[d] | + |
| Penicillic acid | 50 | v. rapid | v. rapid[e] | +++ |
| Pent-2-enoic acid δ-lactone | 50 | 0·05 | 0·0 | N.D. |
| Patulin (clavacin) | 30 | 30 | 0·0 | +++ |
| Parasorbic acid (Hex-2-enoic acid δ-lactone | >20 | 0 | 0 | ++ |
| Citraconic anhydride | >16 | N.D. | 1·06[d] | N.D. |
| Itaconic anhydride | >15 | N.D. | 0·18[d] | N.D. |
| Succinic anhydride | > 7 | N.D. | 0·16[d] | ++ |
| Methyl protoanemonin | 3·5 | 0·5 | 0 | ++ |
| β-Propiolactone | 3·3 | 3·8 | 0·004 | +++ |
| β-Angelica lactone | 2·2 | 0·05 | 0 | + |
| Hex-2-enoic γ-lactone | 2·0 | 0 | 0 | + |
| Acrylonitrile | 1·5 | 0 | 0 | N.D. |
| Ethyleneimine | 0·6 | 0 | 0 | + |
| Propylene oxide | 0·3 | 0 | 0 | + |
| 5-Hydroxymethyl furfural | 0·3 | 0·25 | 0 | N.D. |
| Sodium maleate | 0·26 | 0 | 0 | − |
| Ethylene oxide | 0·18 | 0 | 0 | − |
| Vinylene carbonate | 0·17 | 0·02 | 0 | +++ |
| Coumalic acid | 0·15 | 0·015 | 0 | − |
| Styrene oxide | 0·15 | 0 | 0 | N.D. |
| α-Angelica lactone | 0·12 | 1·1 | 0 | − |
| Glycidol | 0·10 | 0 | 0 | N.D. |
| αβ-Dimethyl maleic anhydride | 0·08 | 0 | 0 | + |
| Penicillin G | 0·07 | 1·3 | 0 | + |
| Coumarin | 0·06 | 0 | 0 | (−)[c] |
| 6-Aminopenicillanic acid | 0·04 | 0·15 | 0 | + |
| Hex-3-enoic acid γ-lactone | 0·022 | 0·5 | 0 | − |
| Citraconic acid | 0·017 | 0 | 0 | N.D. |
| L-Ascorbic acid | 0·015 | 0 | 0 | N.D. |
| Sorbic acid | 0·015 | 0 | 0 | N.D. |
| Dehydroacetic acid | 0·015 | 0·01 | 0 | N.D. |
| Sarkomycin | 0·012 | 0 | 0 | + |
| Hex-4-enoic acid γ-lactone | 0·01 | 0·55 | 0 | + |
| α-Methyl tetronic acid | 0·01 | 0 | 0 | + |
| Aflatoxins ($B_1$ + $G_1$) | 0·008 | 0 | 0 | ++++ |
| γ-Butyrolactone | 0·005 | 0 | 0 | − |
| Maleic hydrazide | 0·002 | 0·05 | 0 | + |
| Itaconic acid | 0 | 0 | 0 | N.D. |
| Bovolactonol | 0 | 0 | 0 | N.D. |
| D-isoascorbic acid | 0·01 | 0 | 0 | N.D. |
| " Maple Lactone " | 0 | 0 | 0 | N.D. |
| γ-Nonalactone | 0 | 0 | 0 | N.D. |
| Aesculin | 0 | 0 | 0 | N.D. |

*Footnotes to Table I.*

[a] Acidic substances were first cautiously neutralized for use in the kinetic tests.

[b] References to carcinogenic activity are from Dickens and Jones (1961, 1963*a*, 1963*b*, 1965), unless otherwise indicated in the text, and mainly refer to sarcoma induction after repeated subcutaneous injection at a single site.

[c] Material was toxic in the animal tests.

[d] Rivett and Sidgwick (1910).

[e] Penicillic acid, open-chain form, is in tautomeric equilibrium with the cyclic lactonic form.

[f] This compound is chemically 3-methyl cyclopentane-1,2-dione and is not a lactone.

N.D. = Not determined.

(Table I ; cf. the slow addition reaction for maleic acid first observed by Morgan and Friedmann, 1938).

The penicillin G-cysteine interaction has been studied by Nakken, Eldgarn and Pihl (1960) who report reaction constants higher than those found here, but they used a different buffer (phosphate) and their measurement was indirect, based upon the change of optical activity of the penicillin-cysteine mixture. In our experiments, the appearance of an acidic group, which is a much more rapid reaction than the loss of SH-group, does not seem to be consistent with a mechanism proposed for the recation by these authors' (mechanism " I ", on p. 90 of Nakken *et al.*, 1960). On the contrary it seems possible that an unstable thioester might first have been formed by cleavage of the 4-membered ring, and this could spontaneously hydrolyse to penicilloic acid, accounting for the acidic group formed, and to free cysteine, accounting for the relatively small loss of SH-group observed by us. Whether this is the real course of this complex reaction remains to be studied.

If one considers the upper portion of Table I, indicating the more highly SH-reacting compounds, the following members have been shown to be capable of tumour induction in animals : maleic anhydride, penicillic acid, patulin, phenyl vinyl ketone, parasorbic acid, succinic anhydride, $\beta$-propiolactone, 2-hexenoic $\gamma$-lactone, $\beta$-angelica lactone, methyl protoanemonin, vinylene carbonate (Dickens and Jones, 1961, 1963*a*, 1965). All of these carcinogenic compounds have a value of the reaction constant with cysteine, $k_2$, equal to or greater than 0·17. But there is not much correlation between the approximate Carcinogenic Index of the biologically active compounds and their relative chemical reactivity in this reaction ; for example vinylene carbonate ($k_2 = 0{\cdot}17$) is about as active a carcinogen as maleic anhydride, penicillic acid, patulin or phenyl vinyl ketone, for which substances $k_2 = 70$, 50, 30 and 200 respectively (Dickens and Jones, 1961, 1963*a*, 1965). We have obtained no tumour in rats with *N*-ethyl maleimide or sodium maleate, which are chemically sufficiently reactive to fall into this group, though the former compound was too toxic for a clear-cut result in our test. Compounds of high chemical reactivity which we are now testing for carcinogenicity in mice, including 2-pentenoic acid $\delta$-lactone, the higher homologue of which is the naturally occurring substance parasorbic acid (2-hexenoic acid $\delta$-lactone) which is marked carcinogenic (Dickens and Jones, 1963*a*). This pentenoic acid $\delta$-lactone is reported (Haynes 1948, p. 48, citing Sir Robert Robinson and P. B. Medawar, private communication) to be about twice as active as the hexenoic acid $\delta$-lactone as a selective inhibitor of tissue growth *in vitro*. Ethyleneimine itself has been reported as probably carcinogenic and many substituted ethylene imines are known carcinogens (Walpole, Roberts, Rose, Hendry and Homer, 1954). Propylene oxide (even in aqueous solution) but not ethylene oxide, was reported to be carcinogenic in the rat (Walpole, 1958). We have found no tests of carcinogenicity of acrylonitrile or of 5-hydroxymethyl furfural and are at present testing the latter substance and also itaconic anhydride in mice. Citraconic anhydride remains to be tested.

If we now consider those compounds in Table I which react more slowly ($k_2$ below 0·17) with the SH group of cysteine, it is true that many of these are either quite weak, slow-acting carcinogens ($\alpha\beta$-dimethyl maleic anhydride, penicillin G, 6-amino-penicillanic acid, 4-hexenoic acid lactone, $\alpha$-methyl tetronic acid, maleic hydrazide) or else have not proved to be carcinogenic in our tests

(coumalic acid, $\alpha$-angelica lactone, coumarin, 3-hexenoic $\gamma$-lactone, $\gamma$-butyrolactone ; Dickens and Jones, 1961, 1963*a*, 1963*b*, 1965). Styrene oxide failed to give skin tumours in mice (Van Duuren, Nelson, Orris, Palmes and Schmitt, 1963). Most of the other compounds in Table I have not yet been tested for carcinogenic activity, although tests are in progress in our laboratory on L-ascorbic acid, isoascorbic acid, sorbic acid, dehydroacetic acid (negative when fed, Spencer *et al.*, 1950), " maple lactone " and $\gamma$-nonalactone.

A striking exception to this attempt to correlate carcinogenic with chemical reactivity in this series of compounds is provided by aflatoxin. This is biologically by far the most active lactone in the whole series. Given subcutaneously to rats, twice weekly doses of as little as 10 $\mu$g. of the mixed purified aflatoxins $B_1$ and $G_1$ have induced sarcomas at the injection site in all the treated rats within 21 to 41 weeks, and even doses of 2 $\mu$g. were carcinogenic, though the induction period was increased to 44 weeks for the appearance of the first tumour. In mice, 10 $\mu$g. doses showed very closely similar carcinogenic activity to 10 $\mu$g. doses in the rat (Dickens and Jones, 1963*b*, 1965). While the value ($k_2 = 0{\cdot}008$) of the reaction constant with cysteine shown in Table I for the mixed aflatoxins $B_1 + G_1$ is probably too low, due to the low solubility of the toxin, it is hardly likely that the true value is enormously greater. The reaction was carried out with vigorous shaking not only in aqueous solution but also in 50 per cent ethanol, in which solvent the yellow colour of the solution showed that a fair proportion of the toxin had dissolved. No appreciable evolution of acid occurred either with or without the addition of cysteine.

Tests of carcinogenicity in the rat with the separated aflatoxins $B_1$ and $G_1$ have shown that the former is the more active, though both components are carcinogens (Dickens and Jones, 1965). The available amounts of the separated toxins (kindly provided by the Tropical Products Institute, London) were insufficient for their separate chemical testing of reactivity in these experiments.

As has been tentatively suggested by Al-Kassab, Davis and Boyland (1963), it is possible that carcinogenic lactones and sulphydryl compounds (these authors used glutathione) might react enzymically in the body tissues. These authors consider that the thioester of hydroxypropionic acid is the probable product of interaction of $\beta$-propiolactone and glutathione, whereas we had previously isolated the chemical reaction-product of $\beta$-propiolactone with cysteine, which proved to be not the thioester but the thioether, *S*-2-carboxyethyl-L-cysteine (Dickens and Jones, 1961). If these supposed differences between the enzymic and chemical types of reaction are confirmed, they may prove to be important.

In any event, a great deal of chemical work requires to be done on the addition of thiol compounds to unsaturated compounds of the type studied in the present work, on which the chemical literature is generally surprisingly uninformative (cf. Dickens, 1964). Table I shows that the more rapid cysteine SH-reactors may be divided into those which are also relatively strong acid producers (patulin, $\beta$-propiolactone) and those which are weak acid producers ($\beta$-angelica lactone, pent-2-enoic $\delta$-lactone, parasorbic acid). Moderate SH-reactors which are relatively strong acid producers, include penicillin, $\alpha$-angelica lactone, hex-4-enoic $\gamma$-lactone, methyl protoanemonin, hex-3-enoic $\gamma$-lactone and 5-hydroxymethyl furfural. Strong acid production may be an indication of the extent of ring-opening by cysteine, but it is difficult to generalize on the present evidence.

SUMMARY

1. A study has been made of the rates of hydrolysis, and rates of chemical interaction with the sulphydryl group of cysteine, of a total of 47 carcinogenic and non-carcinogenic lactones and related compounds. These experiments were made at 25° C. and at neutral pH, in a bicarbonate medium buffered with 5 per cent $CO_2$ in nitrogen.

2. The rates of hydrolysis bore no simple relation to carcinogenic activity in this series.

3. Many of those compounds which reacted fairly rapidly with cysteine (reaction constant, $k_2$, equal to or greater than 0·17 lit. mole$^{-1}$ min$^{-1}$) had proved to be carcinogenic in earlier tests by Dickens and Jones. Exceptions included *N*-ethyl maleimide which however was highly toxic to the animals used.

4. Compounds in this series which showed lower reactivity with cysteine were mainly of lower carcinogenic activity, or were non-carcinogenic. A striking exception was aflatoxin, which, though it is a lactone, may perhaps also owe its very high carcinogenic activity to the presence of other chemical features of the molecule. It reacted only slowly with the sulphydryl group of cysteine under the conditions of our experiments.

We wish to thank Dr. D. K. Black of this Institute for kindly preparing or purifying some of the compounds used in these tests.

This work was supported by a block grant made to the Medical School by the British Empire Cancer Campaign for Research, which we gratefully acknowledge.

### REFERENCES

Brookes, P. and Lawley, P. D.—(1961) *J. chem. Soc.*, 3923.

Al-Kassab, Suad, Davies, W. and Boyland, E.—(1963) *Rep. Brit. Emp. Cancer Campgn*, **40**, 59.

Dickens, F.—(1964) *Brit. med. Bull.*, **20**, 96.

*Idem* and Jones, H. E. H.—(1961 *Brit. J. Cancer*, **15**, 85.—(1963*a*) *Ibid.*, **17**, 100.—(1963*b*) *Ibid.*, **17**, 691.—(1965) *Ibid.*, **19** (in press).

Diez, M. J. F., Osuga, D. T. and Feeney, R. E.—(1964) *Arch. Biochem. Biophys.*, **107**, 449.

Ellman, G. L.—(1959) *Ibid.*, **82**, 70.

Gregory, J. D.—(1955) *J. Amer. chem. Soc.*, **77**, 3922.

Haynes, L. J.—(1948) *Quart. Rev. chem. Soc., Lond.*, **2**, 46.

*Idem* and Jones, E. R. H.—(1946) *J. chem. Soc.*, 954.

Morgan, E. J. and Friedmann, E.—(1938) *Biochem. J.*, **32**, 733.

Nakken, K. G., Eldjarn, L. and Pihl, A.—(1960) *Biochem. Pharmac.*, **3**, 89.

Rivett, A. C. D. and Sidgwick, N. V.—(1910) *J. chem. Soc.*, 1677.

Roberts, J. J. and Warwick, G. P.—(1962) *Rep. Brit. Emp. Cancer Campgn*, **40**, 16.

Ross, W. C. J.—(1962) " Biological alkylating agents ", London (Butterworth).

Spencer, H. C., Rowe, V. K. and McCollister, D. D.—(1950) *J. Pharmacol.*, **99**, 57.

Van Duuren, B. L., Nelson, N., Orris, L., Palmes, E. D. and Schmitt, F. L.—(1963) *J. nat. Cancer Inst.*, **31**, 41.

Walpole, A. L., Roberts, D. C., Rose, F. L., Hendry, J. A. and Homer, R. F.—(1954) *Brit. J. Pharmac. Chemother.*, **9**, 306.

Walpole, A. L.—(1958) *Ann. N.Y. Acad. Sci.*, **68**, 750.